# Why the dual origins of high grade serous ovarian cancer matter

**DOI:** 10.1038/s41467-020-15089-z

**Published:** 2020-03-05

**Authors:** Emily K. Colvin, Viive M. Howell

**Affiliations:** 1 Bill Walsh Translational Cancer Research Laboratory, Kolling Institute, Northern Sydney Local Health District, St Leonards, Sydney, NSW 2065 Australia; 2 Northern Clinical School, Faculty of Medicine and Health, University of Sydney, Sydney, NSW 2006 Australia

**Keywords:** Cancer models, Ovarian cancer

## Abstract

Utilising identical genetic aberrations but targeting different cells, Zhang and colleagues seek to uncover how the cell of origin influences high-grade serous ovarian cancer biology, metastasis and response to treatment.

## Challenges of identifying the cell of origin

Identification of the cell of origin of high-grade serous ovarian cancer (HGSOC) has been a major challenge. The disease is generally diagnosed after the tumour has metastasised throughout the peritoneal cavity^1^, confounding identification of the originating cell type. This has impeded the development of physiologically relevant genetically engineered mouse models (GEMMs). Historically, the two main contenders for the HGSOC cell of origin are the ovarian surface epithelium (OSE) and more recently the fallopian tube epithelium (FTE). Just how the cell of origin impacts the development and progression of HGSOC has not been investigated thoroughly until now. The recent study by Zhang et al.^2^ demonstrates, using mouse models and tumour organoids, that the cell of origin significantly impacts HGSOC growth, metastasis and the HGSOC transcriptome. Compellingly, they also show the cell of origin may play an important role in the response to first-line chemotherapy.

## Evidence from early lesions

Originally, ovarian cancers were thought to arise from OSE that had undergone metaplasia to Müllerian duct-like epithelia before progressing to HGSOC^3^. However, direct evidence of this is limited and no bona fide precursor lesions have been observed in the ovary^4^. The FTE was originally identified as a strong contender for the cell of origin of HGSOC in *BRCA1/2* mutation carriers who underwent risk-reducing salpingo-oophorectomy^5^. Examination of the fallopian tubes from these women demonstrated the presence of proliferative lesions now designated as serous tubal intra-epithelial carcinomas (STICs)^6^. STICs have subsequently been shown to also occur in non-*BRCA1/2*-associated HGSOCs, often contain identical *TP53* mutations as their associated tumours and are now considered a potential precursor lesion of HGSOC^7^. However, experimental evidence that identifies the cell-of-origin as either arising from the OSE or FTE is often contentious, with current evidence suggesting that both the FTE and OSE could give rise to HGSOC^8,9^. Determining the cell of origin of HGSOC is necessary for the development of better in vitro and in vivo models of early carcinogenesis, studying prevention strategies, improving early detection and may also be important for determining optimal treatment strategies.

## Limitations of GEMMs

While direct experimental evidence demonstrating the definitive cell of origin of HGSOC is lacking, gene expression studies using human FTE, OSE and HGSOC samples reveal that the majority of HGSOC more closely resemble FTE, although a significant proportion of HGSOC share similar expression patterns to the OSE^8,9^. Importantly, these studies highlight the likelihood that HGSOC may arise from more than one cell type. However, demonstrating this using GEMMs has proven difficult due to the lack of OSE-specific promoters. Early attempts to model HGSOC in mice occurred when the prevalent opinion was that tumours arise from the OSE. Conditional genetic alterations were introduced into the OSE either using *Amhr2-cre* transgenic mice or by directly injecting adenoviral Cre recombinase under the ovarian bursa^10^. While these methods were able to target the desired genetic changes to the OSE, they are not OSE-specific; *Amhr2* is expressed throughout the entire Müllerian duct and after injection under the ovarian bursa there is also the high possibility of exposure of adenovirus in the mouse oviduct. Furthermore, not all models developed using these techniques resulted in tumours resembling HGSOC, with some mice developing tumours resembling other ovarian cancer subtypes. These limitations make it difficult to deduce that the OSE represents the cell-of-origin of HGSOC. Following the identification of the FTE as a potential cell of origin for HGSOC, mouse models were developed targeting mutations to the mouse FTE using the *Pax8*^11^ or *Ovgp1*^12^ promoters to drive Cre recombinase expression. Depending on the targeted genes, several of these models demonstrated the development of STICs as well as ovarian tumours with HGSOC features. However, due to the limitations of specifically targeting the OSE, there have been no direct comparisons to demonstrate the difference the cell of origin makes to the evolution of HGSOC.

## Direct comparison of contenders for the cell of origin

The study by Zhang et al. comprehensively unravels the key differences the cell of origin can have on the course of HGSOC. By using strategies that target identical genetic aberrations in the FTE and the OSE, the authors were able to compare differences in tumour development, metastasis and response to therapy. HGSOC is characterised by virtually pathognomonic *TP53* mutation and aberrations in the *RB* family occur in up to 67% of patients^13^. To target *RB* inactivation (using T121) and/or *Trp53* mutations to the FTE, Zhang et al. used the established Pax8rtTA;TetOCre mice, which have previously been used to model HGSOC in mice with some success^11^. To target the same genetic aberrations to the OSE, they used *Lgr5Cre*^*ERT2*^ mice, and verified that when activated in adult mice *Lgr5* is restricted to the OSE and absent in the FTE. *Lgr5* was originally discovered in the stem cells of the intestine and colon, but its expression has also been demonstrated in the OSE and FTE^14^. However, *Lgr5* has not previously been used to establish OSE-driven mouse models of HGSOC. A summary of the phenotypes seen in the different mouse models used is shown in Table 1. Their results support numerous other published studies showing multiple genetic aberrations are required to induce HGSOC in mice^10^. This study demonstrates *Lgr5* can be used to target the OSE to induce HGSOC in mice and this model represents a mouse model of HGSOC derived exclusively from the OSE.

**Table 1:** Phenotypes of genetically engineered mouse models targeting genetic lesions to the OSE and FTE by Zhang et al.^2^.

Genetic lesion	Targeting Lgr5+ OSE	Targeting Pax8+ FTE
*Trp53* ^*R172H*^ hemizygous	No tumours	No tumours
RB family inactivation (*T121*)	HGSOC Low penetrance (8%), Long latency (18 months)	STICs Low penetrance (55%) EARLY LETHAL^a^ 100% by 2 months
*Trp53* ^*R172H*^ hemizygous; *T121*	HGSOC 100% at 11 months	STICs 100% at 1 month 88% ovarian metastasis EARLY LETHAL^a^ 100% by 2 months

^a^EARLY LETHAL phenotype due to thymic involvement.

## Organoids provide a new approach

**Fig. 1:**
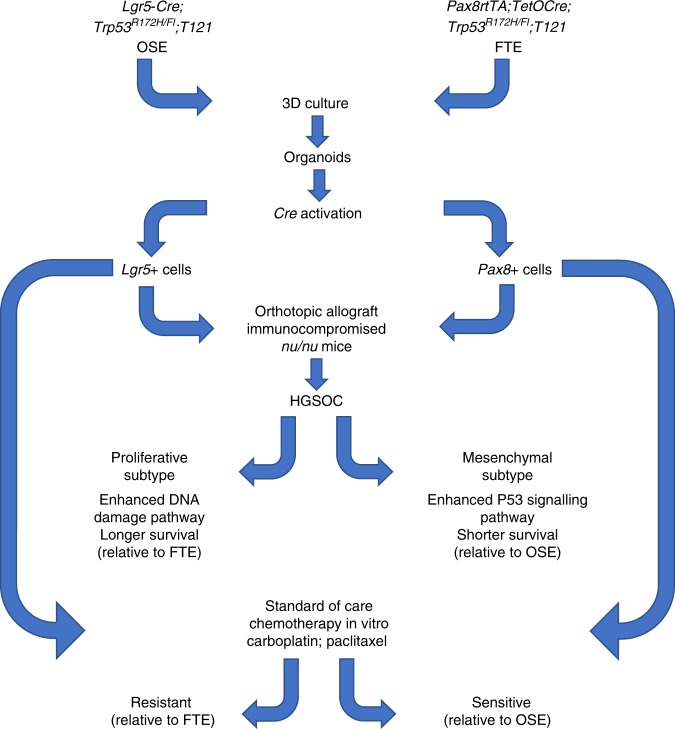
The same genetic lesions targeted to OSE and FTE organoids result in different HGSOC-subtype in mice and different sensitivities to standard of care chemotherapy in vitro.

Even though Zhang et al. were successful in targeting genetic aberrations to the FTE or OSE, they were unable to directly compare differences in HGSOC progression in FTE-derived and OSE-derived mouse models. This was due to *Pax8rtTA;TetOcre;Tp53*^*R172H/fl*^*;T121* mice dying early from enlarged thymi, found to be due to leakiness of the *TetOcre* in the thymic epithelium. To circumvent this, the authors established organoids from the FTE or the OSE of the GEMMs and then induced the genetic aberrations in vitro, before transplanting the organoids into recipient mice to allow them to form tumours. Their approach is depicted in Fig. 1. This allowed the authors to directly compare the effects of the cell of origin on the subsequent development of tumours. FTE-derived organoids formed tumours that grew faster and were more likely to metastasise than their OSE counterparts. The differences seen in vivo were further supported by gene expression differences between OSE and FTE-derived tumours. These results nicely demonstrate that despite identical driver mutations, downstream tumour development is greatly affected by the cell of origin. FTE-derived tumours showed enhanced p53 signalling, while OSE tumours showed enriched DNA repair pathways. When compared to human data from The Cancer Genome Atlas, OSE-derived tumours more closely resembled HGSOC of the proliferative subtype, while FTE-tumours were more similar to mesenchymal-type HGSOC. One of the most interesting findings from this study from a clinical perspective is that there were marked differences in response to the current standard-of-care therapies for HGSOC, with OSE-derived organoids being more resistant to carboplatin and paclitaxel.

## Implications

The development of reliable, highly penetrant HGSOC GEMMs that faithfully recapitulate the human disease from the pre-invasive stages all the way through to metastatic disease has been largely impeded by the lack of highly specific and reliable methods to target the candidate cell of origins (i.e. the OSE and the FTE), rather than a lack of knowledge as to the best genetic drivers to use. Even the promoters used in this study are not highly specific to the OSE or the FTE. *Pax8* is also expressed in the endometrial and renal tubular epithelium and the *Pax8* promoter has previously been used to generate mouse models of renal cancer^15^. *Lgr5* is expressed in the stem cells of the intestine and although the authors mention no intestinal carcinomas were found in *Lgr5-Cre;Tp53*^*R172H/fl*^*;T121* mice, future use of the *Lgr5* promoter to develop OSE-derived mouse models of HGSOC should be approached with caution as any genetic mutations being studied in the OSE will also be altered in the intestines. The limitations of the chosen promoters, combined with the leakiness seen from the TetOcre mice seen in this study, make the use of organoids an attractive alternative as it bypasses the possible oncogenic changes that may be seen in other organs. The use of organoids has steadily been increasing in recent years as a more physiologically relevant way to study cancer in vitro. Zhang et al. also showed that organoids can be transplanted into recipient mice to generate tumours that are able to recapitulate many of the features of HGSOC including the development of metastases and ascites.

Zhang et al. provide strong evidence that the cell of origin plays an important part in the progression of HGSOC and may influence treatment response. They also highlight the potential of using organoids for the development of more reliable, efficient and cost-effective ways to model HGSOC in the mouse. With the improvement of gene editing technologies, it is anticipated that inducing tumorigenic organoids from human FTE or OSE is the next step in understanding the impact of the cell of origin on the initiation and progression of HGSOC.
